# A novel closed reduction technique for treating femoral shaft fractures with intramedullary nails: Kirschner wire and the fulcrum technique

**DOI:** 10.1097/MD.0000000000045614

**Published:** 2025-11-07

**Authors:** Wangsheng Wu, Huajuan Wang, Weijun Huang, Qunyang Zheng, Jie Yu, Bingsheng Liu

**Affiliations:** a The Quzhou Affiliated Hospital of Wenzhou Medical University, Quzhou People’s Hospital, Quzhou, Zhejiang, PR China; b Department of Orthopedic, Quzhou Kaihua Second People’s Hospital, Quzhou, Zhejiang, PR China.

**Keywords:** closed reduction, femoral shaft fracture, fulcrum technique, intramedullary nailing

## Abstract

Femoral shaft fractures (FSF) are usually treated with closed reduction and intramedullary nailing. In this study, we report a novel technique with Kirschner wire to achieve closed reduction. From January 2020 to January 2023, this technique was used on 32 patients with FSF (AO 32 A and 32 B). The fracture was reduced with a radiolucent traction table, 3.0 mm Kirschner wire and the fulcrum technique was conducted. The operative effect was evaluated on the basis of the operative time, reduction time, fluoroscopy time, and intraoperative blood loss. All 32 patients were treated by closed reduction. The mean operative time was 70.4 ± 12.3 minutes (range, 55–90 minutes), of which 15.2 ± 5.6 minutes (range 10–25 minutes) was used for fracture reduction. The mean intraoperative fluoroscopy times were 18.8 ± 4.6 times (range 15–26 times). The mean hemoglobin loss was 1.56 ± 0.84 g/dL (range 1.2–2.0 g/dL). The injured limb obtained excellent alignment after intramedullary nailing. All patients successfully completed a follow-up after fracture healing. The healing time ranged from 3 to 7 months. Displaced FSF (AO 32 A and 32 B) in adults can be treated by the fulcrum technique in a closed fashion and intramedullary nail fixation. This technique is easy to perform, does not increase the cost, can quickly obtain effective reduction, reduce the operation time and intraoperative fluoroscopy times, protect the blood supply of the fracture site, minimize the blood loss, and leads to excellent fracture healing.

## 1. Introduction

Femoral shaft fractures (FSF) are a common orthopedic injury.^[1]^ Closed reduction can reduce the damage to the blood supply at the fracture site and preserve the fracture hematoma which is rich in growth factors and vitally important for fracture healing.^[2]^ With a high rate of union and minimal complications, intramedullary nailing is generally regarded as gold standard treatment for most FSF.^[3,4]^ Closed reduction relies on indirect fracture reduction with the use of fluoroscopy, so the key step of the operation is to obtain a satisfactory closed reduction. The femur is the hardest bone in the human body, and it also has a lot of muscle attachment. Orthopaedic traction tables can effectively correct shortening, rotation and angular displacement of fractures, but they are often ineffective in correcting lateral displacement, which is the main reason for the difficulty in successful closed reduction. Successful closed reduction and effective maintenance of reduction is a challenge, which often requires repeated reduction, resulting in long surgical time and prolonged radiation exposure and sometimes open reduction, which will inevitably increase the damage to the soft tissue surrounding the fracture site, disrupt the blood supply at the fracture end, and affect the natural process of fracture healing.^[5–8]^ In addition, open reduction will lead to a significant increase in intraoperative bleeding and increase the risk of postoperative infection. Some surgeons applied a new type of reduction device, and some surgeons used a robot-assisted reset procedure to achieve rapid and effective fracture reduction with minimal invasive.^[9,10]^ But these operations required additional expensive instruments, and required a certain learning curve. In order to achieve rapid, effective and convenient reduction, we propose to use Kirschner wire and the fulcrum technique to correct lateral displacement and improve the success rate of closed reduction.

## 2. Materials and methods

Our study was reviewed and approved by the Medical Ethical Committee and IRB of Quzhou People’s Hospital and the informed consents have been obtained from all patients for publication. A total of 32 patients were included in the present retrospective study and were treated surgically with this technique from January 2020 to January 2023. The inclusion criteria for the patients were as follows: Displaced femoral shaft fracture (AO/OTA 32 A and 32 B); age > 18; fresh fractures (within 2 weeks after injury); closed injuries; at least 1 year follow-up. The exclusion criteria were: pathological fractures; revision surgeries; accompanied by proximal or distal fractures of the ipsilateral femur; local skin infection before injury; previous injury to the femur; patients with severe osteoporosis. All procedures were performed by the same group of 3 surgeons. The 32 patients had an average age of 42 (range 18–66) years and included 20 males and 12 females. According to the AO/OTA classification system, there were eighteen 32 A cases, fourteen 32 B cases. Regarding laterality, 21 patients had fractures on the left side, and 11 patients had fractures on the right side. The interval from admission to the day of surgery was approximately 2 to 6 days with an average interval of 4.3 days.

### 2.1. Operative technique with case demonstration

**Figure 1.**
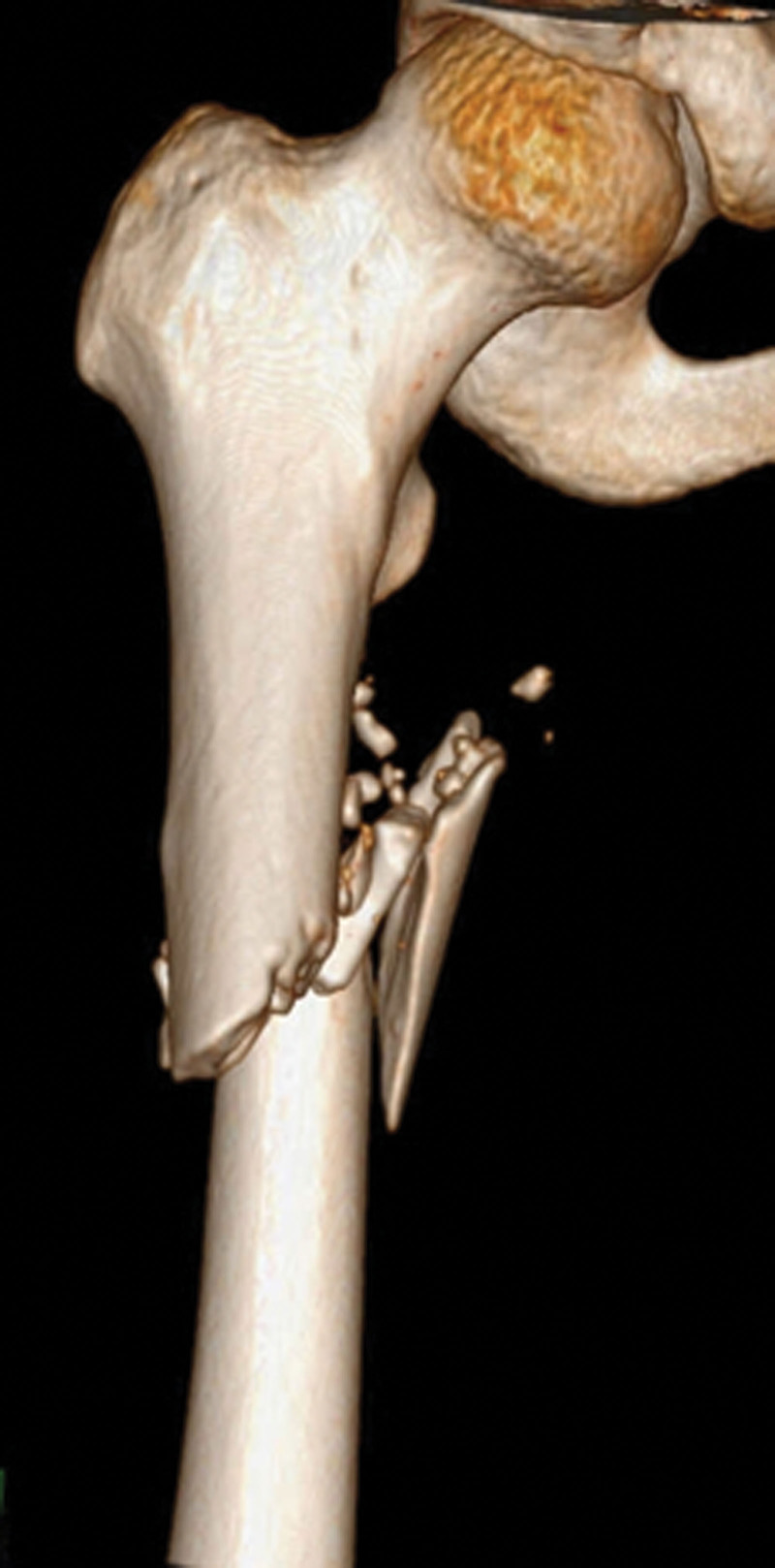
A 20-year-old male sustained a femoral shaft fracture (AO/OTA 32 B) after a traffic accident.

**Figure 2.**
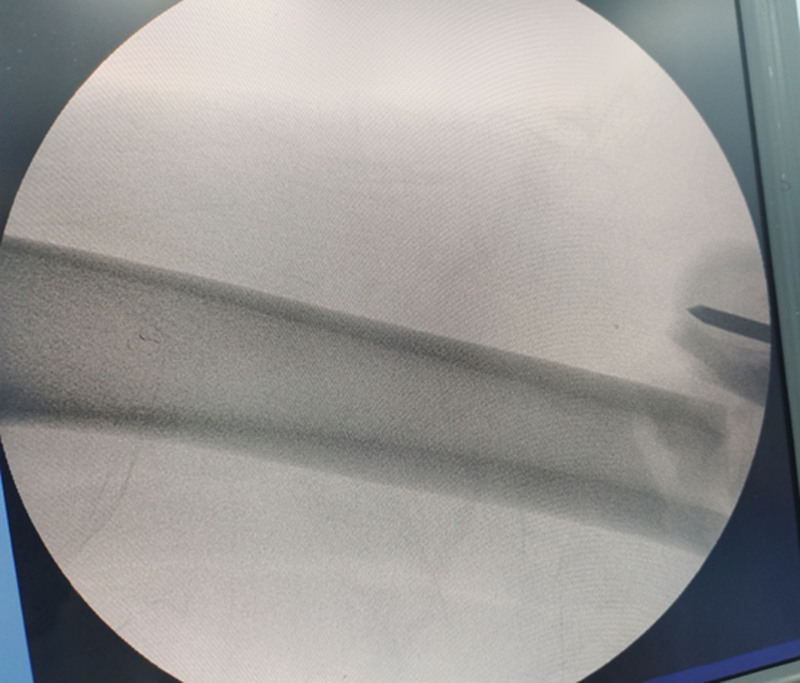
Intraoperative lateral radiograph showed the length recovery after traction, but there was anterior-posterior lateral displacement.

**Figure 3.**
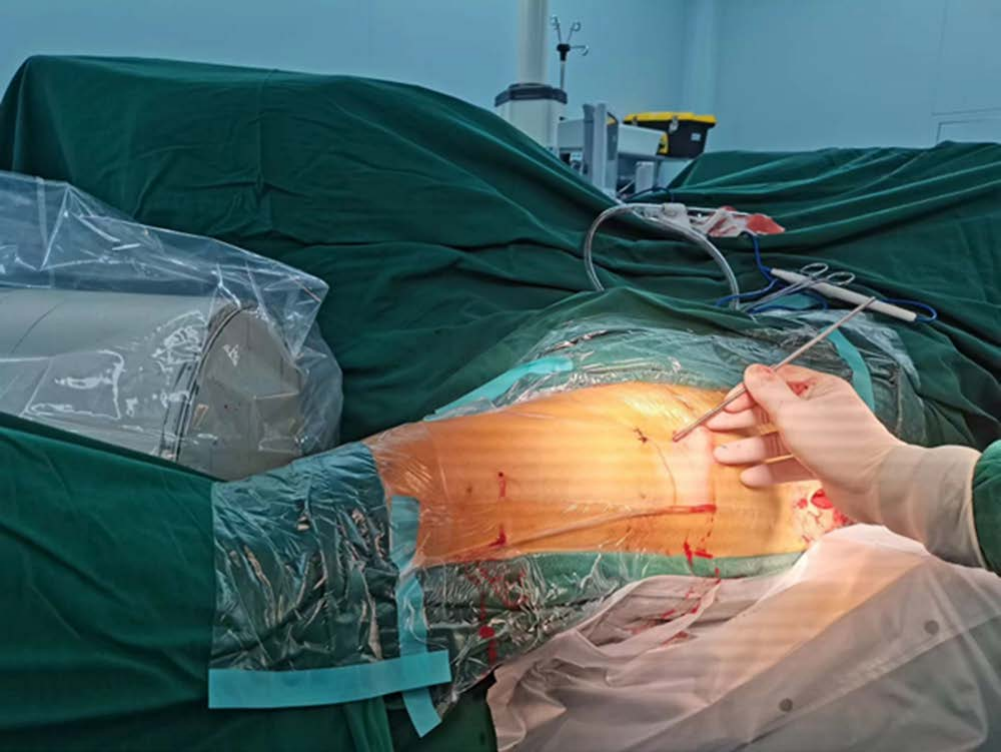
A 3.0 mm Kirschner wire was inserted percutaneous from anterior to posterior.

**Figure 4.**
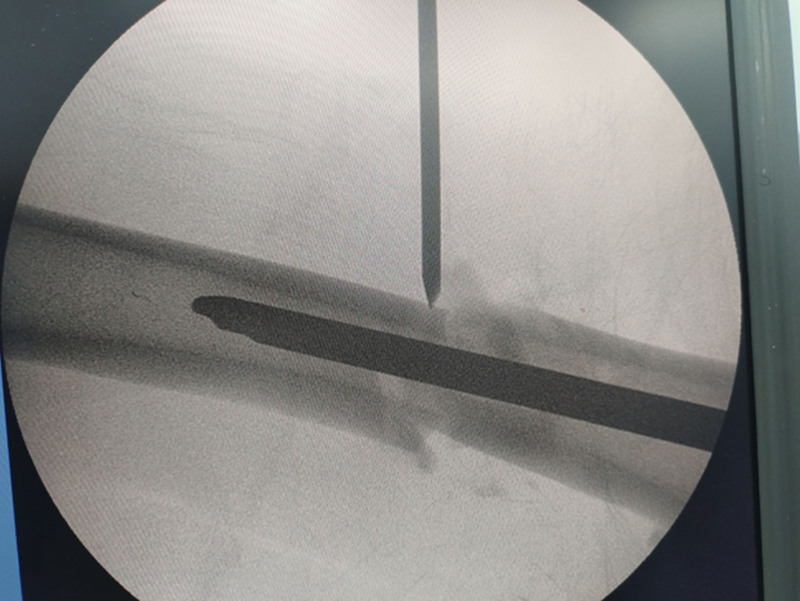
Proximal fracture fragment was used as the fulcrum to pry the distal fracture fragment to restore the alignment of the femoral.

**Figure 5.**
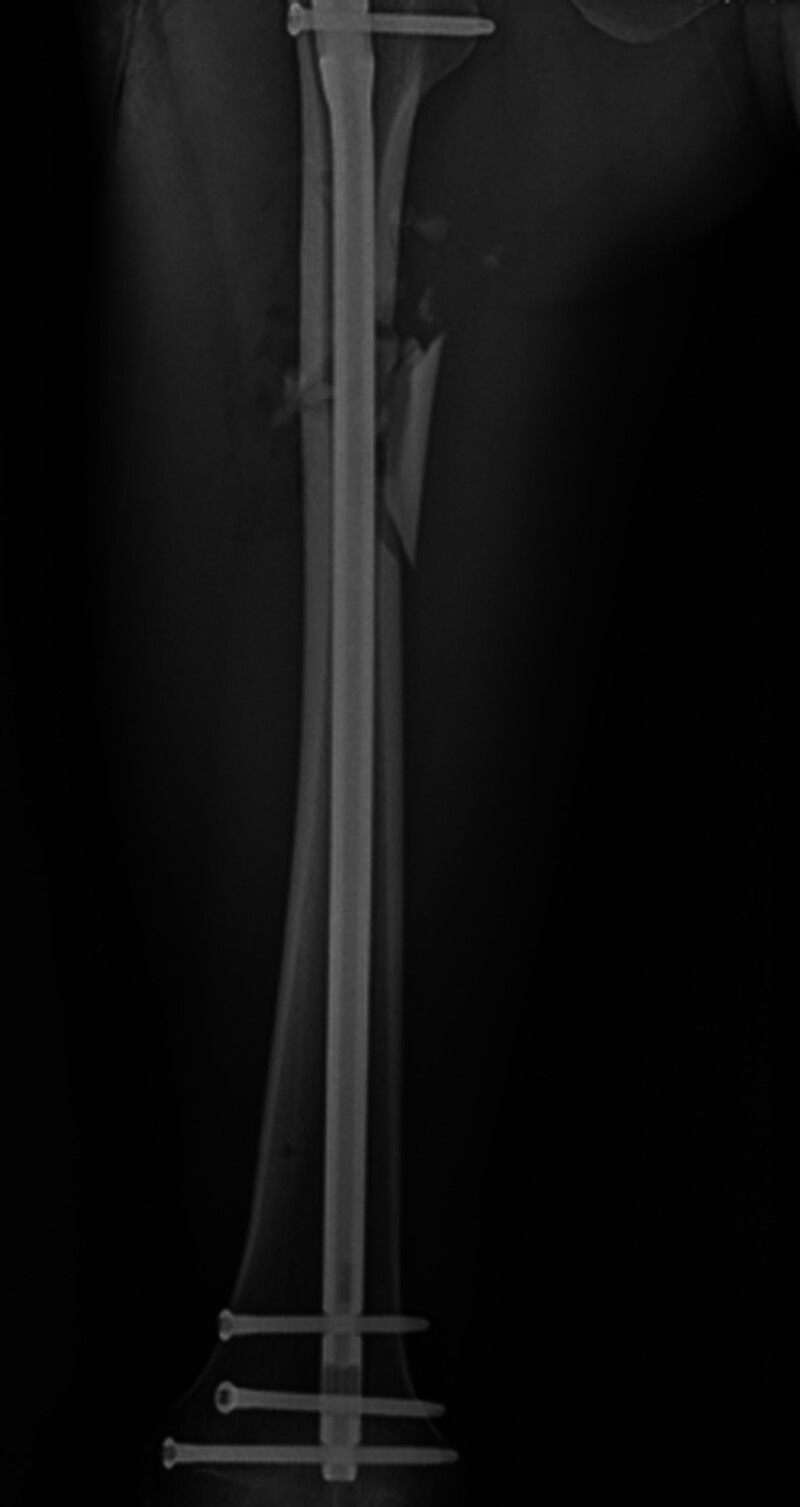
Postoperative x-ray showed satisfactory reduction.

**Figure 6.**
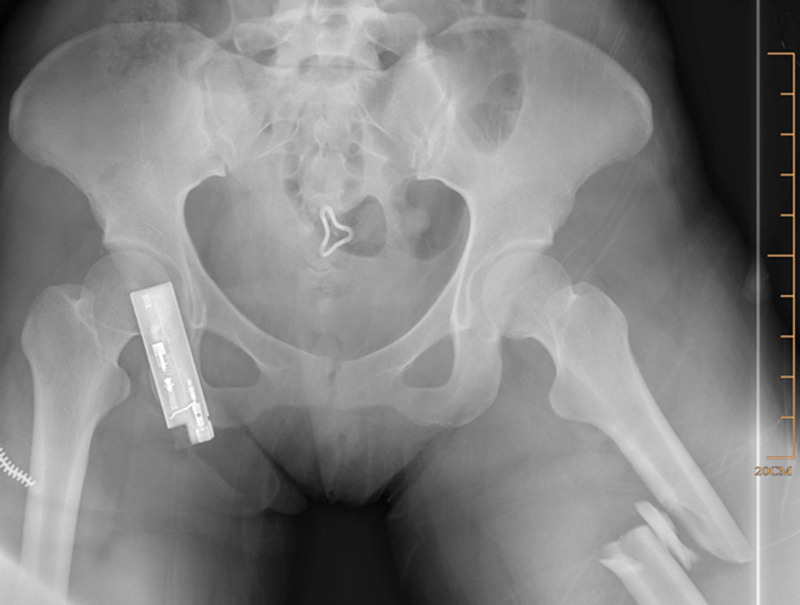
A 32-year-old female sustained a femoral shaft fracture (AO/OTA 32 B) after a traffic accident.

**Figure 7.**
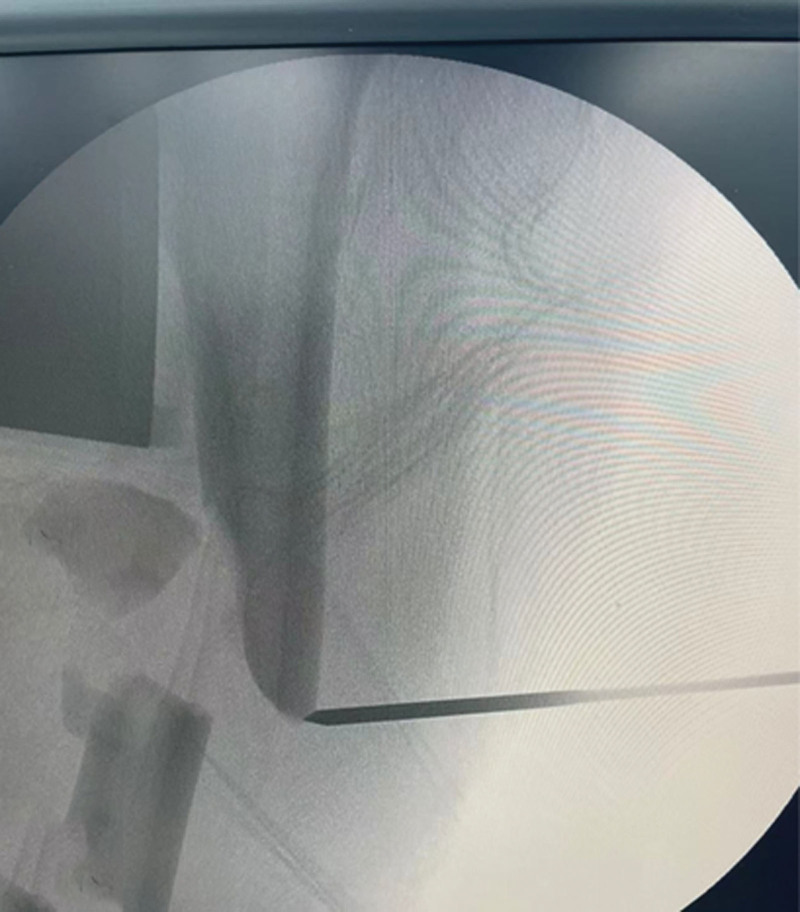
Intraoperative anteroposterior radiograph showed the length recovery after traction, but there was medial-lateral displacement.

**Figure 8.**
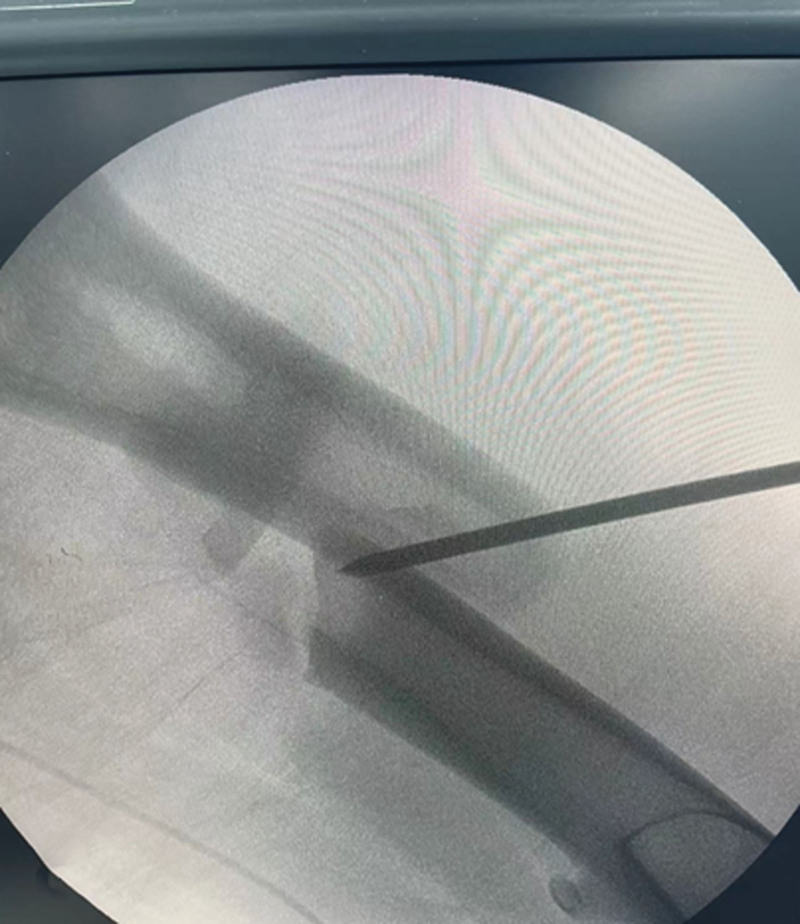
A 3.0 mm Kirschner wire was inserted from lateral to medial through the fracture site.

**Figure 9.**
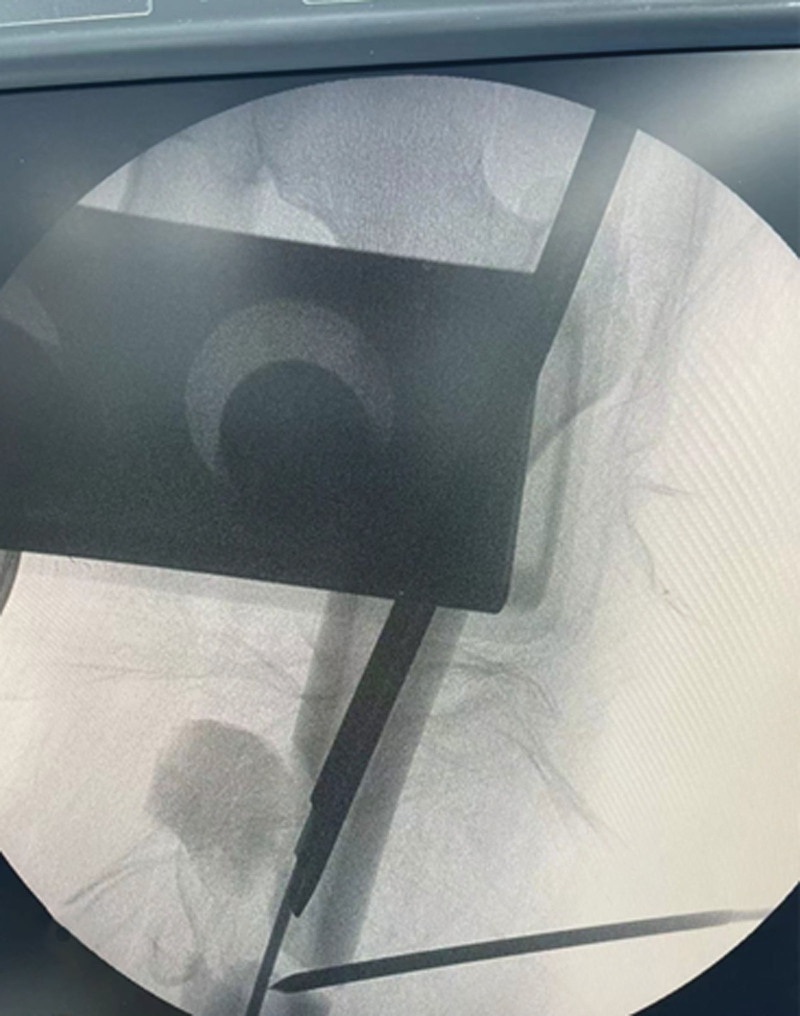
The proximal fracture fragment was used as the fulcrum to pry the distal fracture fragment to restore the alignment of the femoral.

**Figure 10.**
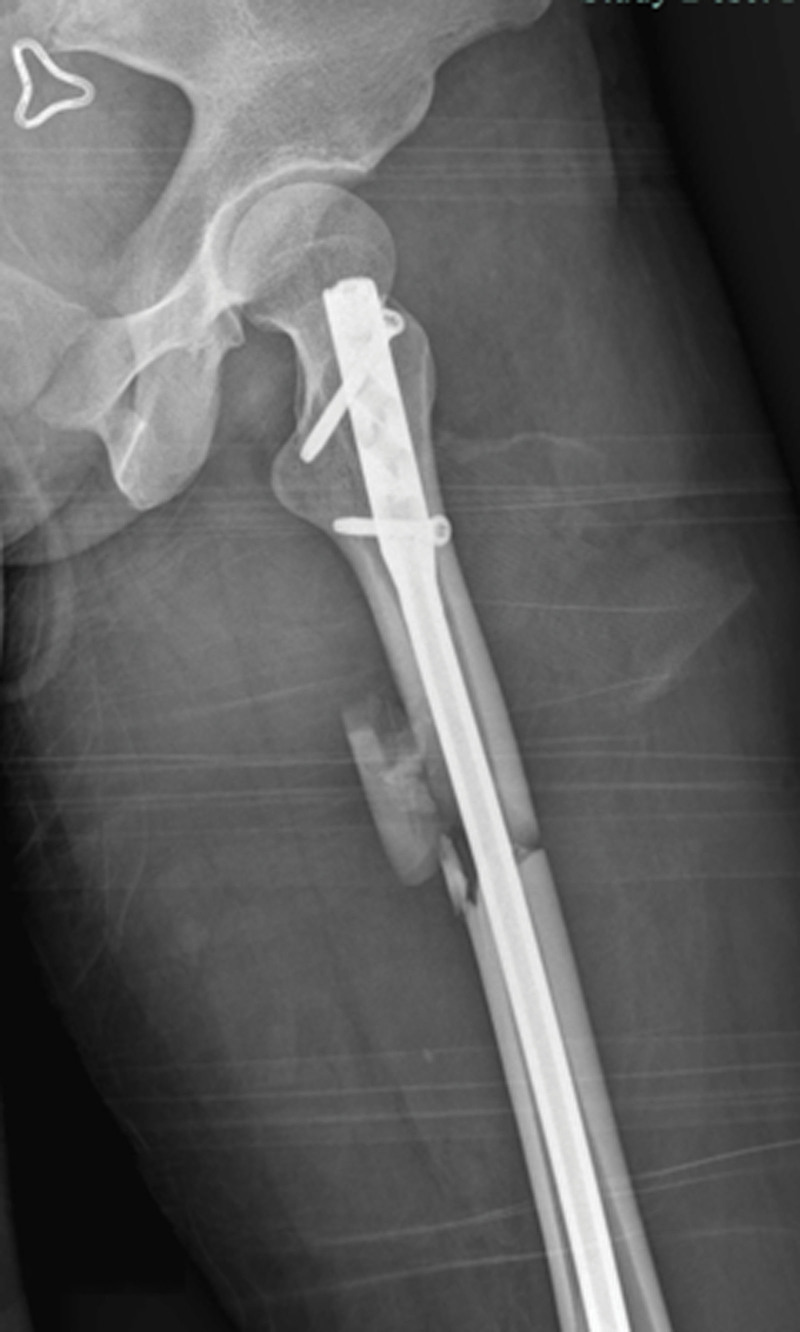
Postoperative x-ray showed satisfactory reduction.

The patient was positioned supine on a radiolucent traction table with the affected limb extended, then adduct the affected limb and gradually increase the traction force to correct shortening, angulation and rotation under the guidance of C-arm fluoroscopy. Usually, only lateral displacement remains after the above procedure. Then the leg was prepared and draped. The fracture site was located by fluoroscopy and a 3.0 mm Kirschner wire was inserted from the point. If there is anteroposterior displacement, the orientation of the Kirschner wire is from anterior to posterior; if there is medial-lateral displacement, the orientation of the Kirschner wire is from lateral to medial. For example, in case 1, a 20-year-old male sustained a FSF (AO/OTA 32 B) after a traffic accident and was operated on the fourth day after the injury (Fig. 1). The length recovered after traction, but there was anterior-posterior lateral displacement (Fig. 2). A 3.0 mm Kirschner wire was inserted from anterior to posterior through the fracture site (Fig. 3). During the insertion of the Kirschner wire, when the proximal fracture fragment was touched, the Kirschner wire was slid through the distal end to explore the distal fracture fragment. After the distal fracture fragment is touched, the Kirschner wire continues to move forward about 1 cm, and then the proximal fracture fragment was used as the fulcrum to pry the distal fracture fragment to restore the alignment of the femoral (Fig. 4). Then inserted the finger reduction tool, the insertion and fixation procedures for the IM nail were performed using the standard method (Fig. 5). In case 2, a 32-year-old female sustained a femoral shaft fracture (AO/OTA 32 B) after a traffic accident and was operated on the third day after the injury (Fig. 6). The length recovered after traction, but there was medial-lateral displacement (Fig. 7). A 3.0 mm Kirschner wire was inserted from lateral to medial through the fracture site (Fig. 8). During the insertion of the Kirschner wire, when the proximal fracture fragment was touched, the Kirschner wire was slid through the distal end to explore the distal fracture fragment. After the distal fracture fragment is touched, the Kirschner wire continues to move forward about 1 cm, and then the proximal fracture fragment was used as the fulcrum to pry the distal fracture fragment to restore the alignment of the femoral (Fig. 9). Then inserted the finger reduction tool, the insertion and fixation procedures for the IM nail were performed using the standard method (Fig. 10).

### 2.2. Postoperative management

The second day after surgery, the patients were instructed to perform passive function exercises of the affected limb. If possible, patients were encouraged to gradually start weight-bearing exercises with an assistive device 1 week after the operation.

## 3. Results

### 3.1. Surgical outcomes

All 32 cases of displaced FSF (AO 32 A and AO 32 B) were reduced in a closed fashion. After IM nailing, the injured limb acquires the excellent alignment. The mean operative time was 70.4 ± 12.3 minutes (range 55–90 minutes), of which 15.2 ± 5.6 minutes (range 10–25 minutes) was used for fracture reduction. The mean intraoperative fluoroscopy times were 18.8 ± 4.6 times (range 15–26 times). The mean hemoglobin loss was 1.56 ± 0.84 g/dL (range 1.2–2.0 g/dL).

### 3.2. Follow-up findings

All patients were followed up. The average follow-up time was 14.6 months (range 12–18 months). During the follow-up, all patients exhibited fracture healing; the healing time ranged from 3 to 7 months. Deep venous thrombosis, breakage of internal fixation, malunion and infection were not observed.

## 4. Discussion

Fractures of the femoral shaft are common high-energy injuries of the lower extremities.^[11]^ Surgery is the first choice in the treatment of adult FSF. Intramedullary nail is the gold standard for FSF, with good union results and a success rate up to 95%.^[12]^ And closed nailing is preferred because of shorter healing time.^[13]^

**Figure 11.**
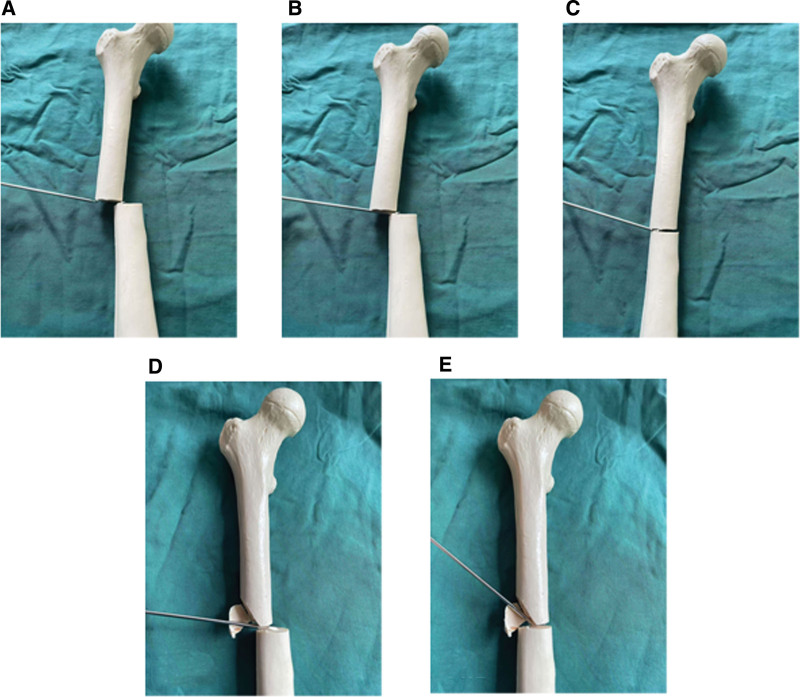
A 3.0-Kirschner wire was inserted from lateral to medial to explore the end of proximal fragment and distal fragment. When the end of the distal fragment was reached, Kirschner wire continued to move 1cm forward in the fracture gap, and then used the proximal fragment as the fulcrum to pry the distal fragment to restore the alignment (A–C). This technique still works well when the comminution area is in the side of fulcrum (D–E).

The Kirschner wire fulcrum technique proposed in this study can achieve safe, simple, economical and effective reduction. This technique is only suitable for FSF (AO 32 A and AO 32 B; Fig. 11), because in 32 C the fulcrum cannot be found due to the middle segment. Shui et al proposed hemostatic forceps technology to assist closed reduction, in which 1 vessel clamp was inserted into each distal and proximal fracture fragment, and then muscle tissue was used as the fulcrum to pry up or press down the fracture fragment to achieve reduction.^[14]^ Compared with this technique, only one Kirschner wire was inserted at the fracture end in this study, which resulted in less trauma. In addition, in this study, the more rigid fracture end was used as the fulcrum to make the reduction easier. Rohilla et al proposed the Schanz screw technique to assist closed reduction, in which a Schanz screw is inserted into the cortex of the distal fracture and then the reduction is achieved by lifting or pressing the Schanz screw.^[15]^ Compared with the insertion of a threaded Schanz screw, the insertion of an unthreaded Kirschner wire in this study caused less damage to soft tissue; In addition, the Kirschner wire in this study did not need to penetrate the bone cortex, and the damage to the bone would be smaller. Thakur et al proposed bone levers technique to assist closed reduction, but the results of this study showed that the nonunion rate was as high as 10.3%, and 5.2% of patients showed superficial infection.^[16]^ In this study, all patients achieved bone healing and no infection occurred. Jiang et al designed a bone traction retractor to assist reduction through transverse bone traction technology.^[17]^ McFerran et al reported that the use of a femoral distractor can safely and effectively assist the reduction of femoral fractures.^[18]^ In contrast, the technology used in this study requires no additional expensive equipment, and is simple to operate without cumbersome equipment installation processes. Shewring et al proposed the F-Clamp technique to achieve reduction by lifting and pressing proximal and distal fracture fragments in vitro.^[19]^ In this study, the Kirschner wire used 1 fracture end as the fulcrum to pry the contralateral fracture end, which can achieve more accurate reduction than in vitro operation.

The technique needs to be used with the help of an orthopedic traction table. Although the traction table is not effective in restore the alignment, it can restore the length of the lower limb by longitudinal traction. The recovery of limb length, even excessive traction, is a prerequisite for subsequent operations. It is important to determine the direction of lateral displacement when using this technique. The orientation of Kirschner wire placement is determined by the displacement direction of the fracture. The displacement of fracture fragment in femoral shaft is affected by both the violent direction and the muscle group attached to the fracture fragment. Common displacement includes:

Situation 1. Posterior displacement of the distal fracture fragment, and;
Situation 2. Lateral displacement of the distal fracture fragment.

The orientation of the Kirschner wire is from anterior to posterior in situation 1 and from lateral to medial in situation 2. Finding the fulcrum in this technique is critical. The proximal fracture fragment is usually used as the fulcrum, but the distal fracture fragment is used as the fulcrum if the distal fracture fragment is lateral or anterior. The diameter of the Kirschner wire is usually 3.0 mm, a larger diameter causes more trauma and is difficult to pass through the fragments, the smaller diameter cannot complete the prying function due to insufficient stiffness.

Limitations of this study include its retrospective nature, the small sample size and incomplete data collection.

In conclusion, traction can effectively correct the shortening, rotation and angular displacement, but cannot correct lateral displacement. Our fulcrum technique can be a safe and effective method to correct lateral displacement to obtain a satisfactory closed reduction. The procedure is quick with no need to transfer the patient, and it requires no specialized equipment. It is worthy of clinical promotion.

Our study was reviewed and approved by the Medical Ethical Committee and IRB of our hospitals and the informed consents have been obtained from all patients for publication.

## Author contributions

**Conceptualization:** Wangsheng Wu, Bingsheng Liu.

**Data curation:** Huajuan Wang, Jie Yu, Bingsheng Liu.

**Formal analysis:** Wangsheng Wu, Huajuan Wang, Bingsheng Liu.

**Investigation:** Wangsheng Wu, Huajuan Wang, Weijun Huang, Qunyang Zheng.

**Methodology:** Huajuan Wang, Weijun Huang.

**Supervision:** Bingsheng Liu.

**Validation:** Huajuan Wang, Bingsheng Liu.

**Writing – original draft:** Wangsheng Wu, Huajuan Wang.

**Writing – review & editing:** Wangsheng Wu, Weijun Huang, Qunyang Zheng, Bingsheng Liu.
